# Refractory Anemia with Ring Sideroblasts Associated with Marked Thrombocytosis Complicated by Massive Splenomegaly Treated with Lenalidomide Resulting in Resolution of Splenomegaly but Severe and Prolonged Pancytopenia

**DOI:** 10.1155/2013/718480

**Published:** 2013-04-07

**Authors:** Gordon Taylor, Dominic Culligan, Mark A. Vickers

**Affiliations:** Department of Haematology, Aberdeen Royal Infirmary, Aberdeen AB25 2ZN, UK

## Abstract

Refractory anemia with ring sideroblasts associated with marked thrombocytosis (RARS-T) is a hematological malignancy that combines features of both a myeloproliferative and myelodysplastic disorder. There have been recent reports of the successful treatment of anemia in 2 patients with RARS-T with lenalidomide. Here we report the successful treatment of massive splenomegaly in a patient with a long history of RARS-T resulting in complete resolution of splenomegaly, but with prolonged severe cytopenias. We also report the acquisition of the t(3;12)(q26;p13) translocation previously described in cases of myelodysplasia and the potential for transformation to myelofibrosis.

## 1. Case Presentation

**Figure 1:**
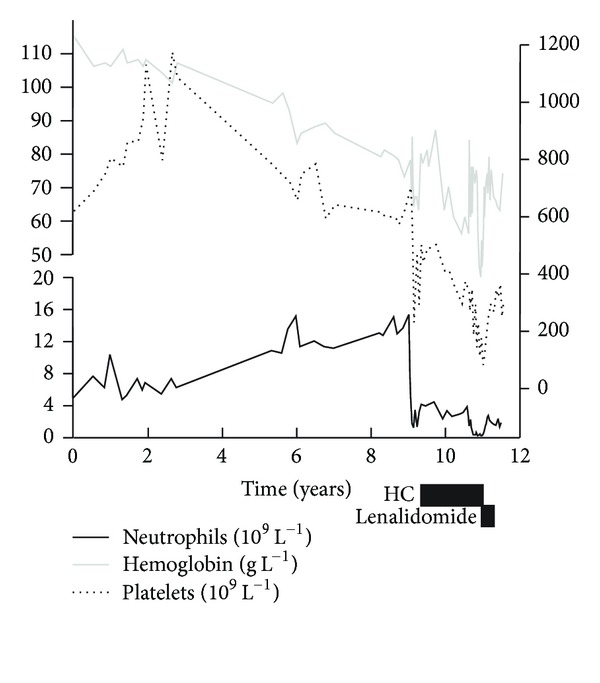
Table demonstrating neutrophil count, platelet count, and pretransfusion hemoglobin against time from diagnosis up until the time of transplant, also showing the effect of hydroxycarbamide and lenalidomide on these.

A 47-year-old female presented 12 years ago with migrianous headaches, a persistent marked thrombocytosis (greater than 700 × 10^9^/L), and 25% bone marrow ring sideroblasts. Bone marrow cytogenetics showed a normal 46XX karyotype. The subsequent development of anemia with dyserythropoiesis, increasing percentage of ring sideroblasts to 45%, and confirmation of the presence of the *JAK2V617F* mutation allowed the diagnosis to be confirmed as the recently characterised refractory anemia with ring sideroblasts associated with marked thrombocytosis (RARS-T). With disease progression she developed massive splenomegaly extending past her umbilicus. Splenomegaly was unsuccessfully treated with hydroxycarbamide, since doses large enough to cause a minor reduction in spleen size resulted in worsening of anemia requiring red cell transfusion. Following the report during 2010 of two recently diagnosed patients with RARS-T showing an improvement in anemia when treated with lenalidomide, treatment was changed to lenalidomide 10 mg daily, taken for 21 days out of every 28 day cycle. Within 2 weeks of starting lenalidomide the patient became pancytopenic and her transfusion requirement increased. After completing a total of 4 monthly cycles of lenalidomide, she remained heavily transfusion dependent (requiring 2 to 3 units of red cells every 1-2 weeks) and had persistent National Cancer Institute common toxicity criteria grade 4 neutropenia and grades 2-3 thrombocytopenia. Lenalidomide treatment was therefore stopped. During the treatment period her spleen had started to soften and over the following 2 months off treatment there was complete resolution of her palpable massive splenomegaly. She remained profoundly pancytopenic and red cell transfusion dependent (Figure 1).

**Figure 2:**
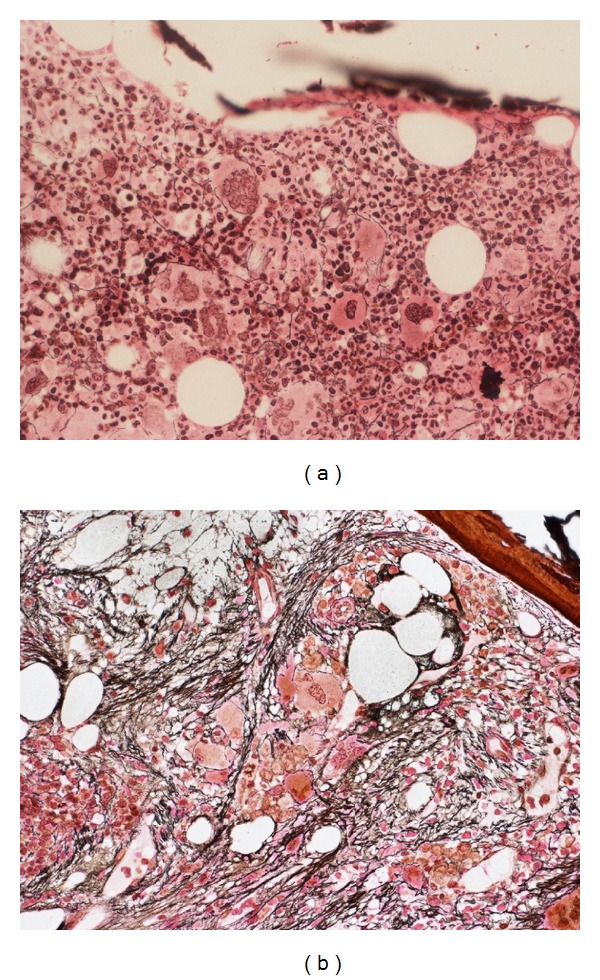
(a) demonstrates the patient's bone marrow reticulin stain at initial presentation demonstrating only fine reticulin deposition around megakaryocytes. (b) is bone marrow biopsy 10 years after diagnosis demonstrating grade 3 reticulin fibrosis.

A bone marrow biopsy sample was taken 6 weeks after stopping lenalidomide. The trephine biopsy revealed clusters of dysplastic megakaryocytes and erythroid cells as before, but now with frank marrow fibrosis (grade 3) that had not been present at diagnosis (Figures 2(a) and 2(b)). 

Four months after stopping lenalidomide her spleen had grown back to past her umbilicus, her transfusion frequency was reduced, her neutrophil count had recovered to greater than 1 × 10^9^/L, and her platelet count had returned to within normal range. The patient underwent an unrelated donor hematopoietic stem cell transplant using reduced intensity conditioning of fludarabine, melphalan, and alemtuzumab. Following an immediate posttransplant period of poor engraftment, with donor chimerism of not more than 30%, the graft was lost with progression of massive splenomegaly and increasing transfusion dependence. Repeat bone marrow biopsy 125 days posttransplant identified the presence of a t(3;12)(q26;p13) translocation involving *ETV6* and *EVI1,* not previously identified in this patient. This translocation has previously been described in cases of acute myeloid leukemia, myelodysplastic syndromes (MDS), and chronic myeloid leukemia. The patient received splenic radiotherapy for control of pain and started treatment with the combined *Jak1* and *Jak2* inhibitor ruxolitinib but did not respond to this and subsequently died from sepsis. 

## 2. Discussion

RARS-T is a provisional World Health Organization recognised disease entity amongst the group of myeloproliferative/myelodysplastic crossover syndromes [1] and is associated with the presence of the *Jak2V617F* mutation [2], *Tet2* mutations, and less commonly the *MPLW515K/L* mutation [3]. These mutations probably contribute to the myeloproliferative component of the disease. Recently described *SF3B1* mutations appear to be associated with myeloid neoplasms with ring sideroblasts [4, 5]. Lenalidomide is an immunomodulatory thalidomide analogue (IMiD) that has been shown to have some efficacy in the treatment of anemia in other myelodysplastic syndromes [6, 7], particularly when associated with deletions of chromosome 5q, where transfusion independence occurs in some two-thirds of patients [7]. Lenalidomide with prednisolone has also been reported to be efficacious in improving anaemia and splenomegaly in idiopathic myelofibrosis although treatment has been limited by high rates of grades 3-4 hematological toxicity [8]. 

The two original cases reported by Huls et al. [9] detailing improvements in anemia in patients with RARS-T treated with lenalidomide, importantly, did not appear to have had significant marrow fibrosis at the time of initiation of lenalidomide therapy. In our case, described here, lenalidomide was used at an advanced stage of the disease, when there was clearly extensive marrow fibrosis and massive splenomegaly. The response to lenalidomide in this situation was interesting in that there was complete resolution of the massive splenomegaly but profound worsening of the cytopenias. The likely explanation for this is that the extramedullary hematopoiesis in the spleen is predominantly arising from the malignant *JAK2V617F *mutated clone. Lenalidomide-induced suppression of the clone led to effective resolution of splenomegaly but there is insufficient normal residual hematopoiesis in the fibrotic marrow to allow for any hematopoietic recovery at this late stage in the natural history of RARS-T. In many ways this is akin to the myelosuppression that predicts response to lenalidomide therapy in MDS with del 5q and is thought to result from direct suppression of the 5q-clone but in this instance occurs in the context of sufficient functional hematopoiesis to allow for blood count recovery. A reduction in the detectable* JAK2V617F*-positive malignant clone was also reported in the 2 cases of RARS-T treated with lenalidomide by Huls et al. [9].

Thalidomide with and without prednisolone has been reported to produce responses in primary and secondary MF with improvement in anemia and splenomegaly; however long term treatment is often limited by significant rates of peripheral neuropathy [10]. Pomalidomide is a newer IMiD that does not appear to cause peripheral neuropathy and appears to cause less hematological toxicity than lenalidomide. There are reports of this being effective in the treatment of anemia and splenomegaly in patients with MF [11]. At the time of writing pomalidomide is not licensed for use in the UK. Although combined *Jak1* and *Jak2* inhibitors appear to be effective in reducing spleen size and treating systemic symptoms in patients with primary MF and secondary MF, they appear to cause significant hematological toxicity, particularly worsening of anemia in around a quarter to a third of patients treated [12]. 

## 3. Conclusion

In conclusion, we describe the natural history of a young woman with RARS-T over nearly 12 years and confirm that marked marrow fibrosis and splenomegaly can occur. The pattern of response to lenalidomide should make physicians cautious when considering treatment with IMiDS in patients with RARS-T at an advanced stage of the disease, especially if there is significant bone marrow fibrosis.
